# Obesity-Related Chronic Kidney Disease: Principal Mechanisms and New Approaches in Nutritional Management

**DOI:** 10.3389/fnut.2022.925619

**Published:** 2022-06-24

**Authors:** Alessandra Stasi, Carmela Cosola, Gianvito Caggiano, Maria Teresa Cimmarusti, Rita Palieri, Paola Maria Acquaviva, Gloria Rana, Loreto Gesualdo

**Affiliations:** Renal, Dialysis and Transplantation Unit, Department of Emergency and Organ Transplantation, University of Bari, Bari, Italy

**Keywords:** obesity, chronic kidney disease, nutrition, molecular mechanisms, nutritional strategies

## Abstract

Obesity is the epidemic of our era and its incidence is supposed to increase by more than 30% by 2030. It is commonly defined as a chronic and metabolic disease with an excessive accumulation of body fat in relation to fat-free mass, both in terms of quantity and distribution at specific points on the body. The effects of obesity have an important impact on different clinical areas, particularly endocrinology, cardiology, and nephrology. Indeed, increased rates of obesity have been associated with increased risk of cardiovascular disease (CVD), cancer, type 2 diabetes (T2D), dyslipidemia, hypertension, renal diseases, and neurocognitive impairment. Obesity-related chronic kidney disease (CKD) has been ascribed to intrarenal fat accumulation along the proximal tubule, glomeruli, renal sinus, and around the kidney capsule, and to hemodynamic changes with hyperfiltration, albuminuria, and impaired glomerular filtration rate. In addition, hypertension, dyslipidemia, and diabetes, which arise as a consequence of overweight, contribute to amplifying renal dysfunction in both the native and transplanted kidney. Overall, several mechanisms are closely related to the onset and progression of CKD in the general population, including changes in renal hemodynamics, neurohumoral pathways, renal adiposity, local and systemic inflammation, dysbiosis of microbiota, insulin resistance, and fibrotic process. Unfortunately, there are no clinical practice guidelines for the management of patients with obesity-related CKD. Therefore, dietary management is based on the clinical practice guidelines for the nutritional care of adults with CKD, developed and published by the National Kidney Foundation, Kidney Disease Outcome Quality Initiative and common recommendations for the healthy population. Optimal nutritional management of these patients should follow the guidelines of the Mediterranean diet, which is known to be associated with a lower incidence of CVD and beneficial effects on chronic diseases such as diabetes, obesity, and cognitive health. Mediterranean-style diets are often unsuccessful in promoting efficient weight loss, especially in patients with altered glucose metabolism. For this purpose, this review also discusses the use of non-classical weight loss approaches in CKD, including intermittent fasting and ketogenic diet to contrast the onset and progression of obesity-related CKD.

## Introduction

Overweight and obesity are broadly defined as pathological conditions characterized by abnormal fat accumulation that increases health risks. Since the second half of the last century, the prevalence of obesity has tripled worldwide, reaching approximately 30% in the global population especially in children and adolescents (1).

The phenomenon of obesity has grown tremendously over the last 40 years, thanks to numerous aspects of modern society, often characterized by urbanized areas in which the demand of both high-calories food and junk food exceeds the need for healthy food (2). Moreover, it is wide recognized that unhealthy lifestyles together with sedentary habits and little physical training are closely correlated with an increase in the prevalence of obesity and overweight although there are differences between the female and male gender (3). Additionally, psychological factors such as depression and stress play a role in influencing obesity consolidation (4). Numerous clinical studies have observed that stress promotes weight gain. Especially, the data highlighted that changes in cortisol levels result in alterations of energy balance. Stress-induced cortisol secretion is strongly involved in fat deposition and increases circulating glucose and insulin levels (5). In addition to unhealthy behavioral conditions, many biological factors were found to be mainly associated with the development of overweight. For instance, several studies showed that the alteration in Leptine expression was correlated to severe forms of obesity (6). Further, the alteration in gut microbiota was associated to increased permeability and inflammation of the gastrointestinal tract, which results in systemic inflammation (7). Finally, a large amount of data supports the genetic involvement in obesity. According to these studies, the genetic background of obesity is mainly represented by three groups (i.e., mendelian syndromic obesity, mendelian non-syndromic obesity, and polygenic obesity) regarding the associated gene mutation along with phenotype severity (8).

Although obesity was formerly thought to represent only an independent risk factor for numerous diseases, a large amount of data has now shown that obesity itself represents a chronic and progressive disease strongly influenced by environmental factors (e.g., the availability of high caloric food and low physical activity) and genetic factors. As it is characterized by excessive accumulation of adiposity, obesity is commonly estimated by the body mass index (BMI), calculated as weight (kg)/height (m). Based on this parameter, the World Health Organization (WHO) classifies individuals with a BMI between 25 and 29.9 kg m^–2^ as overweight and those over 30 kg m^–2^ as obese. In addition to BMI, waistline (WC) and waist-to-hip ratio are now important tools to assess fat distribution and contribute to risk stratification. Moreover, obese individuals are classified based on cutoff points of BMI values in obese classes I (30–34.99 kg m^–2^), II (35–39.99 kg m^–2^), and III (>40 kg m^–2^; 1).

In addition to fat accumulation, obesity has been described to be closely associated with an altered metabolic profile. In this scenario, adipose tissue is recognized as a highly metabolically active tissue characterized by an intense secretory profile. Depending on the grade, excess adipose tissue exerts a strong systemic activity resulting in multiorgan involvement, metabolic disorders (i.e., insulin resistance and dyslipidemia), chronic inflammation, and procoagulant state which are recognized as hallmarks of obesity. In addition, obesity strongly affects the onset of several chronic diseases, such as type 2 diabetes (T2D), hypertension, cardiovascular disease (CVD), dyslipidemia, non-alcoholic fatty liver disease, and chronic and acute kidney disease (CKD and AKI). Interestingly, in the past decade, obesity has been recognized as a key contributor in the development and progression of renal failure. The so-called “obesity-related nephropathy” is characterized by several hallmarks such as glomerular hyperfiltration, altered sodium reabsorption, and hypermetabolic demands, which result in decreased renal function (9).

Nutrition professionals should pay special attention to the nutritional status of the obese patient who is losing weight. Weight loss through worsening body composition (i.e., loss of lean mass, thus decreasing basal metabolic rate) indeed should be avoided, as it represents a condition that predisposes the subject to weight regain after dismissing the diet. The need is then felt to explore effective and safe nutritional weight-loss approaches to be applied in obese patients with CKD to improve their cardiometabolic health and to reduce blood pressure (BP) and renal load, eventually allowing them to be on the waiting list for transplantation.

In this review, we will explore nutritional standard strategies for patients with CKD and CKD-obese and the biological aspects related to this complex disease. Finally, we will present the perspective of an integrated approach, which underlies the need for new approaches to improve patient quality of life.

## Effect of Obesity on Chronic Kidney Disease and Its Progression

The increasing burden of obesity has become a widespread public health issue in the adult and child population, rising in epidemic proportion especially in the western world. It is widely suggested that obesity is closely related to CVDs and other metabolic diseases such as hypertension, insulin resistance, T2D mellitus, and chronic inflammation (10, 11). In addition, several large population studies, together with epidemiologic data, observed a strong association between obesity and a high risk of developing and progressing CKD and end-stage renal disease (ESRD; 12, 13).

According to these studies, obesity is predictive of impaired kidney function and represents an independent risk factor for the progression of CKD (14). Furthermore, increased baseline BMI was proven to be an independent predictor of ESRD after adjusting for other comorbidities, such as insulin resistance hypertension and diabetes mellitus. Therefore, evidence suggests that risk of developing CKD is five-fold higher in overweight adults than in the lean population (i.e., BMI > 25 kg/m^2^) and obesity *per se* is a strong predictor of ESRD (15, 16).

In this scenario, many mechanisms have been proposed to elucidate the pathophysiology of obesity-related chronic renal failure (17). Briefly, a combination of hemodynamic factors, mainly hypertension, together with metabolic disorders, exacerbate renal damage in obese patients. Furthermore, visceral adiposity promotes cellular accumulation of free fatty acids (FFA) and triglycerides (TG), leading to oxidative stress and lipotoxicity (18). Additionally, the production of inflammatory mediators, such as adipokines and cytokines, and profibrotic factors increase inflammation, endothelial dysfunction, and renal injury. Ultimately, obesity and CKD share a close association; however, the pathophysiology of obesity-related CKD is seemingly multifactorial (19).

## Pathways Involved in Obesity-Related Kidney Disease

### Pathophysiology of Obesity-Related Chronic Kidney Disease

**FIGURE 1:**
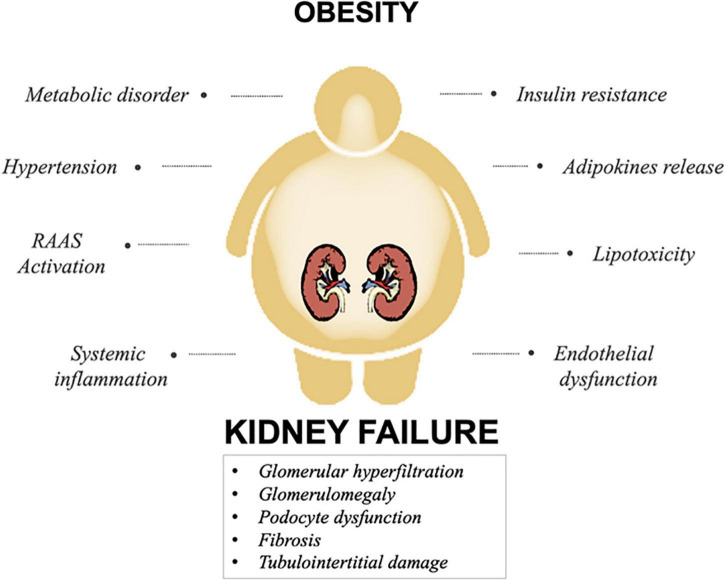
Principal mechanisms involved in obesity-related kidney disease. A combination of several mechanisms involved in obesity, impact on renal function, culminating in kidney damage.

Obesity is tightly correlated to a secondary form of perihilar focal segmental glomerulosclerosis called obesity-related glomerulopathy (ORG). Primarily, the histopathological hallmark of the kidney in obese patients show a glomerulomegaly characterized mainly by mesangial hypertrophy and podocytes dysfunction associated to altered foot process (20, 21). As suggested by several studies, altered renal hemodynamics is a key factor closely linked to renal disorder in overweight patients. Accordingly, hypertension and hyperfiltration mainly contribute to glomerulomegaly and podocyte stress resulting in ORG *via* several mechanisms (22, 23). As result, the hemodynamic change initiates a cascade of events starting with increased glomerular filtration rate (GFR) and intra-glomerular capillary pressure. On the other hand, inflammation, insulin resistance and adipokine dysregulation directly affect renal cells, causing glomerular, and tubulointerstitial damage. Furthermore, intracellular accumulation of FFA and TG in glomerular and tubular cells exerts a lipotoxic activity leading to oxidative stress and apoptosis (24; Figure 1).

### Hemodynamic Factors

The physiological renal response to obesity consists of an increase in renal blood flow, in GFR and tubular reabsorption of sodium (Na). Notably, in several studies, higher BMI is strongly associated with an augmented GFR compared to normal weight renal flow.

Hypertension and hyperfiltration play a pivotal role in obesity-induced renal dysfunction (25, 26). In this scenario, the expansion of renal vascular tautness together with sodium and water retention are the main triggers of glomerular hypertension and hyperfiltration in obesity. Principally, afferent arteriolar vasodilation is the first event driving glomerular hyperfiltration (27). Enhanced renal blood flow due to the afferent vessel dilatation together with efferent vascular contraction intensifies the glomerular filtration resulting in the augmentation of GFR. Otherwise, increased Na channel expression in proximal tubular cells leads to sodium and water reabsorption, exacerbating both afferent vasodilation and glomerular hyperfiltration due to tubuloglomerular feedback (28). Alongside, the overregulation of the renin-angiotensin-aldosterone system (RAAS) play a pivotal role in modulating vasocontraction by increasing sodium retention and hypertension (29). Finally, adipocytes-derived cytokines are also involved in modulating vasoconstriction. For instance, asymmetric dimethyl arginine inhibits nitric oxide production, leading to afferent vasoconstriction. Consequently, the mechanical stress translates into glomerular damage and podocyte dysfunction, detachment, and global glomerulosclerosis (10).

### Renin-Angiotensin-Aldosterone System

Circulating levels of RAAS components are higher in obese individuals than in lean people. In addition, RAAS effectors including aldosterone and Ang II adversely affect CKD progression as they contribute to hypertension and glomerulosclerosis. Along with hypertension, RAAS contributes to endothelial dysfunction *via* multiple mechanisms including hyperleptinemia, and insulin resistance, which represent a key factor in the development of obesity-related CKD (30, 31). Furthermore, Ang II induces intra-glomerular pressure, proteinuria, and stimulates the production of pro-inflammatory and profibrotic factors in renal cells. Moreover, as at the adipose tissue level, RAAS stimulates both adipocyte growth and adipokine production (10). Finally, several data suggest that the overproduction of aldosterone affects insulin and leptin resistance together with the high sodium intake and metabolic dysregulation. Interestingly, according to these studies, a close association between aldosterone levels, BP and salt intake was found (32). It is well-known that aldosterone exerts a role in regulating plasma volume, salt homeostasis, and BP *via* mineralocorticoid receptors expressed in several tissues including adipose tissue. In this scenario, a number of *in vivo* and *in vitro* studies demonstrate that the activation of adipocyte aldosterone receptors regulates adipogenesis and inflammation at the transcriptional level. Further studies demonstrated that mice treated with aldosterone receptor antagonists showed a reduction in reactive oxygen species (ROS) production together with the inhibition of pro-adipogenic and pro-inflammatory genes (33). As observed in numerous studies, in addition to the RAAS activation pathway, aldosterone levels in obese patients seems to be enhanced by several factors including sodium intake and adipose tissue stimulation *via* cytokines (34).

### Inflammation

An increasing number of studies reported the crucial role of obesity-induced inflammation in the development of CKD. Along with the immune system, adipose tissue represents the major source of cytokine secretion due to its endocrine functions (35, 36). Importantly, cytokines could be produced by adipocytes, infiltrating macrophages, or both. Studies have found increased expression of genes related to lipid metabolism (e.g., low density lipoprotein receptor, fatty acid-binding proteins, and sterol regulatory element-binding protein 1), inflammatory cytokines [e.g., tumor necrosis factor α (TNF-α), interleukin 6 (IL-6), and interferon gamma], and insulin resistance [e.g., glucose transporter 1, insulin receptor substrate 1 (IRS1) and Vascular endothelial growth factor (VEGF)] in patients with ORG. Chiefly, TNF-α is one of the main mediators of inflammation that strongly promote insulin resistance. Several data indicated that increased TNF-α levels correlate with the development of glomerulonephritis and tubulointerstitial damage in obese individuals (37). IL-6 is a pro-inflammatory factor produced by adipocytes and immune cells. Many studies suggest IL-6 role in mediating several mechanisms including insulin resistance, RAAS activation, and TGF-β1 pathway regulation. Additional cytokines such as MCP-1, PAI-1, resistin, visfatin, and adipsin (complement factor D) exert a pro-inflammatory activity in obesity-mediated inflammation, which affect kidney function (37, 38).

### Insulin Resistance

Insulin resistance, commonly associated with obesity, is closely linked to renal inflammation and fibrosis *via* numerous molecular pathways (39). Together with inflammation, insulin resistance, contributes to an increased risk of CKD progression by affecting both glomerular hemodynamics and gene expression of profibrotic and pro-inflammatory factors (40).

### Adipokines

Thanks to its endocrine functions, adipose tissue exerts a role in the production of several hormones, cytokines, and chemokines collectively named adipokines. Adipokines are involved in a multitude of processes such as lipid metabolism, insulin resistance, inflammation, atherosclerosis, and extracellular matrix deposition (19). Over and above the widely known mediators TNF-α, IL-6, and MCP-1, the most studied adipokines include leptin and adiponectin. Leptin is a pro-inflammatory adipokine, that is, approximately 5–10 times higher in obese patients and exert a role in controlling appetite and body weight. As suggested by several studies, leptin is involved in the progression of CKD as its expression is correlated with glomerular sclerosis by stimulating mesangial and glomerular hypertrophy (41). Conversely, adiponectin is an anti-inflammatory adipokine, which protects against obesity-related metabolic alterations such as insulin resistance and lipid accumulation. Circulating levels of adiponectin were negatively associated with body fat ratio, reflecting its downregulation in obesity. Interestingly, lower adiponectin levels correlated with higher levels of both proteinuria and GFR in obese patients (38). Finally, others adipocyte-produced cytokines, such as visfatin and resistin, play a crucial role in stimulating RAAS activity and renal inflammation (37, 42).

### Lipid Metabolism and Lipotoxicity

Obesity is characterized by lipid overload associated with ectopic lipid accumulation, which exert a severe toxic effect known as lipotoxicity (43). Notably, kidney lipotoxicity correlated with structural and functional changes of mesangial cells, podocytes, and tubular cells. Furthermore, several data indicated that the accumulation of FFA and TG in the kidney cells results in glomerular and tubulointerstitial damaging (25). According to these notions, lipid accumulation in the kidney appears to promote both cytokine and mitochondrial ROS production exacerbating renal injury. Accordingly, mitochondrial dysfunction, oxidative stress, and inflammation affect mesangial cells, podocytes, and proximal tubular epithelial cells (44).

## Dysbiosis of Gut Microbiota in Obesity-Related Kidney Disease

The human gut microbiota is a dynamic community of continuously evolving microorganisms living in the digestive tract. Factors that influence its composition include age, the host genetic background, dietary preferences, geographical areas of living, and the use of antibiotics, prebiotics, and probiotics (45). The microbiota comprises more than 1,500 species of bacteria, but also viruses and fungi (46). Bacillota, Bacteroidota, Actinomycetota [synonyms for Firmicutes, Bacteroidetes, and Actinobacteria, respectively, (47)], Proteobacteria, Actinobacteria, and Verrucomicrobia are six phyla that dominate the human gastrointestinal tract (48). Among them, Bacillota and Bacteroidota account for around 90% of the gut microbiota (49, 50). Bacteroidota and Actinomycetota are considered good bacteria because they contribute to intestinal health and glucose homeostasis, confer host resistance to infective disease, and synthesize vitamins (51). Human microbiota partially ferment dietary polysaccharides, such as resistant starch, dietary fiber, and other low digestible polysaccharides into short chain fatty acids (SCFAs; 52). Bacteroidota mainly produces acetate and propionate, while Bacillota synthesizes butyrate (53). SCFAs are able to reduce inflammation, regulate intestinal epithelial cells functions modulating their proliferation and differentiation, impact gut motility and strengthen the gut barrier functions, as well as host metabolism (54). Conversely, Bacillota and Proteobacteria can exert a negative influence on glucose and fat metabolism and are therefore considered harmful bacteria (45). A complex symbiosis exists between the human body and its microbiota, modeled by the known plasticity of the gut microbiota; in fact, microbiota composition remains relatively unchanged during acute perturbations, allowing it to rapidly rearrange itself (55). Although chronic exposition to harmful stress factors can profoundly alter the relationship among microorganisms, leading to an imbalanced equilibrium of microbial species known as dysbiosis, which has been often associated with the development of diverse diseases (56). It is known that the dysbiosis can influence the gastrointestinal tract (diarrhea, irritable bowel syndrome; 57), the immune system (allergy, multiple sclerosis, type 1 diabetes, inflammatory bowel diseases, and rheumatoid arthritis; 58), the central nervous system (Alzheimer and Parkinson diseases and autism; 59), and host energy metabolism (obesity, T2D, and atherosclerosis; 60). Unfortunately, it is not yet clear whether dysbiosis can be the cause or consequence of these diseases.

Recently, the role of microbiota has been suggested as a link between obesity and kidney disease. In the last few decades, the gut microbiota has been proposed as an additional factor favoring the obesity condition. Dysbiosis leads to increased fatty acid oxidation, triglyceride storage, and alteration of SCFA proportions, subsequently promoting a high risk of developing other diseases (61, 62). In addition, dysbiosis leads to impairment of the gastrointestinal barrier with the release of endotoxins such as lipopolysaccharides (LPS) into the circulatory system (63) causing metabolic endotoxemia. LPS, the constituents of the outer membrane of Gram-negative bacteria cell, circulates in the plasma to various organs and activates the immune system, stimulating them to secrete numerous pro-inflammatory cytokines, leading to the induction of a low-grade systemic inflammatory response (64) and oxidative stress (65, 66). Obesity-related dysbiosis may be a causative factor inducing the inflammation that affects disease progression and can cause damage in various organs such as the kidney (66). In the literature, obesity and consumption of a high-fat diet are frequently associated with decreased populations of Bacteroidota and increased populations of Bacillota (67). Studies on obese animals and humans (62, 68) suggested that the increase in Bacillota vs. Bacteroidota species happen because the former was more effective in extracting energy from food than the latter, thus promoting a more efficient calorie absorption and subsequent weight gain (69). Turnbaugh et al. observed that the differences in the metagenomic profile of two twins influenced the development of obesity. Indeed, the microbiome of the obese twin was enriched in genes coding for nutrient transporters while that from the lean twin was enriched in genes coding for enzymes associated with carbohydrate metabolism (70). These data suggest that alterations in bacterial composition/diversity are generally associated with changes in the metabolic profile of the microbiota, which also influence host health. Accordingly, in the last decade, the Bacteroidota/Bacillota ratio has been frequently considered as a possible hallmark for obesity (71, 72). Conversely, an inverse or no difference in the Bacteroidota/Bacillota ratio of overweight and normal weight subjects has been observed by other studies (70, 71, 72). In addition to these two phyla, some have observed an abundance of Actinomycetota in obese persons (68). From these observations, obese patients showed lower bacterial diversity than lean subjects, suggesting the existence of other compositional changes at the family, genus, or species level that might be more relevant than the Bacteroidota/Bacillota ratio (73). Advances in next-generation sequencing and bioinformatics tools have shown that this relationship is far more complex than was previously postulated (54). There is growing evidence supporting a role for the gut microbiota in the development and progression of kidney disease in obese patients with dysbiosis (66). Increased gut permeability caused by dysbiosis leads to augmented transfer of accumulated toxins in the blood, which, in turn, contribute to systemic inflammation, release of proinflammatory cytokines, and oxidative stress (74). The presence of dysbiotic gut microbiome is not only associated with enhanced production of harmful metabolites and uremic toxins, but also with the cessation of beneficial metabolites (e.g., SCFA) formation. The progressive reduction of renal excretory function in these patients causes an excessive accumulation of urea and uremic toxins, such as indoxyl sulfate (IS) and p-cresol sulfate (PCS), which have a negative impact on the kidneys and other organs. High urea concentrations can lead to increased gut pH, favoring mucosal irritation and promoting the growth of proteolytic bacteria, thus favoring the maintenance of intestinal dysbiosis and inflammation (75). This could significantly modify the gut microbiota by enhancing the growth of bacterial species capable of metabolizing urea into ammonia (Lachnospiraceae, Enterobacteriaceae, and Ruminococcaceae) and promoting the reduction of species that have an anti-inflammatory renoprotective activity (such as Bacteroidota, Lactobacillus, Prevotellaceae, and Bifidobacterium; 76).

The uremic toxins, IS and PCS, are derived exclusively from protein fermentation by proteolytic bacteria and are normally excreted by the kidneys. As their levels depend on dietary protein absorption and/or intake, a low-protein diet (LPD) has been evaluated as a strategy to delay the progression of kidney disease with discordant results (77, 75). They are classified as protein-bound uremic toxins, as they circulate in the blood tightly bound to albumin (78). Patients affected by CKD, especially those with worsening end-stage disease, cannot efficiently remove uremic toxins, favoring the accumulation of higher levels in the blood. Evidence show that IS damages endothelium, hepatocytes, muscle cells, myocytes, renal proximal tubular cells, and intestinal cells. Furthermore, PCS may negatively affect leukocytes, adipocytes, renal proximal tubular cells, intestinal cells, and myocytes (79).

The effect of obesity and gastrointestinal dysbiosis well correlate with the affection of kidney function by the induction of systemic inflammation. Moreover, the progression of kidney injury and dysfunction could in turn increase the severity of gastrointestinal dysbiosis. Thus, modulation of the gastrointestinal microbiota has been hypothesized as a possible strategy for the management of obesity-related kidney disease.

## Nutritional Treatment of Obesity Resource Identification Initiative

As the risk of morbidity and mortality increases in overweight and obese individuals, weight reduction should be recommended to prevent further progression of obesity risk. The growing consumption of both energy-dense nutrients and low nutritional value foods significantly influence the onset of obesity. Hence, the main goals for obesity treatments should be focused on a reduction of 10% in body weight for obesity classes I and II and >10% for class III. According to the Italian Standard for obesity care, dietary plans for obese individuals need to be revisited with several modifications in carbohydrates, proteins, fats, and fiber amount. Carbohydrates, preferably derived from fiber-rich foods, should be range from 50 to 55% of the total daily kilocalories intake. The percentage of sugar should range between 10 and 12% of daily kilocalories intake. It was also observed that low carbohydrate diets characterized by higher amounts of unsaturated fats resulted in reducing body weight up to 10% and ameliorated both glycemic and lipid profiles. Finally, low-glycemic food intake should be preferred in patients with impaired glucose tolerance to maintain weight loss over time. On the other hand, the protein amount should be up to 15% of the total daily caloric intake, which results in between 0.8 and 1 g/kg dbw/die (dbw indicates “desirable body weight,” or rather the weight corresponding to a BMI of 22.5 kg/m^2^). Additionally, a large amount of evidence showed that a reduced fat intake significantly contributes to weight loss. During obesity, a balanced nutritional plan should be composed of a very low fat intake (<30% of the total daily kcal) associated with reduced cholesterol assumption (≤300 mg/die). Finally, an adequate daily intake of fiber (30 g/die) exert a positive effect on obese individuals by improving metabolic parameters and intestinal functions. In conclusion, nutritional plans should be associated with regular physical activity to reach a weight loss of 10% in 6 months. Moreover, foods of high biological value, such as vegetables, legumes, cereals, and extra virgin olive oil, are an important aspect in control of metabolic waste.

### Nutritional Treatment of Patients With Chronic Kidney Disease

The KDIGO 2012 clinical practice guideline (80) recommends that patients with CKD receive expert dietary advice and a nutritional education program based on achieving four goals: limited introit of daily proteins, adequate caloric daily intake, decreased sodium intake, and controlled or reduced phosphorus and potassium dietary assumption.

### Proteins and Caloric Intake

According to the Kidney Disease Outcome Quality Initiative (KDOQI) Clinical Practice Guideline for Nutrition in CKD, the average protein assumption in adults in Western countries is 1.35 g of proteins/kg bw/die. This nutritional plan leads to a positive nitrogen balance in patients with CKD, resulting in the development of an uremic state and metabolic acidosis. On the contrary, a reduction of protein intake between 0.6 and 0.3 g of proteins/kg bw/day together with the supplementation of keto acid analogs (KA) is strongly recommended in patients with CKD, and it should be supported by sufficient energy intake. Additionally, an adequate caloric diet (≥30 kcal/kg bw/day; 81) associated to the low protein intake can contrast the protein-energy malnutrition and the increase in uremia (82). However, unbalanced nutrition (either in excess or in deficiency) can promote the onset of metabolic acidosis due to the increased level of uremic toxins in the patient’s blood. Moreover, uremia leads to appetite loss, anorexia, nausea, or vomiting, which promotes sarcopenia and reduced bone mass (uremic osteoporosis; 83). To date, four main dietary regimes are the most commonly recommended for the nutritional treatment of CKD:

- *Normal protein diet*: Protein intake of 0.8 g/kg bw/day as the general adult population in patients with G1 to G3a CKD.
- *Low-protein diet*: Protein intake of 0.6 g/kg bw/day (0.4 g/kg of animal origin), in patients with G3b to G5 CKD.
- *Low-protein vegan diet*: Protein: 0.7 g/kg bw/day (from grains and legumes), in patients with G3b to G4 CKD.
- *Supplemented very-low-protein diet (sVLPD)*: Protein: 0.3–0.4 g/kg bw/day supplemented with KAs in patients with G4 to G5 CKD (84).

### Sodium

High levels of sodium in patients with CKD is strongly associated with hypertension and CVD. Moreover, a large amount of data showed that elevated sodium concentration is an independent risk factor for CKD progression (85). In this scenario, according to the WHO, the daily consumption of sodium chloride should not exceed 5–6 g (i.e., 2–2.3 g sodium/day). Notably, a hyposodium diet is not recommended in CKD to avoid sodium depletion, hypotension, and worsening renal function (86).

Educational dietary strategies for sodium intake include a controlled protein diet combined with practical nutritional advice, the DASH diet, and the Mediterranean diet (MD). The DASH-Sodium Trial demonstrated that the DASH diet can reduce BP in obese individuals due to the reduced sodium intake associated to elevated intakes of vegetable and potassium (87). The MD (88) is a traditional style of nutrition, defined by a large consumption of whole grains, vegetables, legumes, and seasonal fruits and a constant and moderate use of nuts and extra virgin olive oil. Moreover, the main protein sources of MD are represented by fish, whereas the consumption of red meat and saturated fatty acids is rare. Both the DASH diet and MD have been widely associated with reduced risks for CV, coronary artery disease, stroke, and T2D (87, 88).

### Phosphorus

Phosphorus is a mineral useful for bone growth, mineralization, and acid-base homeostasis, and its concentration is regulated by kidneys. However, due to progressive loss of function from the stage G3b of CKD [estimated GFR (eGFR) ≤45 ml/min], kidneys reduce their ability to regulate the phosphorus concentration resulting in hyperphosphatemia (81). Interestingly, five RCTs evaluated the effect of LPD or VLPD with KA supplementation on serum phosphate levels in patients affected by CKD G4 and G5. After the intervention, a statistically significant reduction in phosphorus concentration in the patients’ blood was observed (89, 90, 91, 92, 93). In addition, several observations demonstrated that the bone-derived fibroblast growth factor 23 named FGF23 is strongly involved in the regulation of circulating levels of phosphate and vitamin D and seems to play a role in the development of insulin resistance (94). Interestingly, few data highlighted that very-low-protein diet associated with KA supplementation is able to reduce FGF23 levels in patients with CKD (95). Importantly, phosphorus intake control is recommended from the earliest stages of CKD, starting at 700 mg/day to reach further reduction during CKD progression. Based on this concept, for the stage >G3a a daily phosphate intake of 700 mg/day is recommended, while for <G3b phosphate intake should be lowered to 300 mg/day, excluding the most phosphate-rich foods from the diet (96).

### Potassium

Potassium is a cation with the highest concentration in an intracellular compartment of the human body. Its main mechanisms of action are related to cellular electrophysiology, BP, and neuromuscular function. Currently, the LARN guidelines (reference intake of nutrients and energy for Italian population) defines an assumption of 3.9 g/day of potassium for the Italian adult population (97).

However, elevated potassium serum concentration has been associated with various physiological alterations (e.g., ventricular arrhythmias, muscle weakness, hypertension, and death). Furthermore, both homeostasis and blood concentration of potassium are strongly linked to kidney function as potassium excretion decreases with the progression of kidney failure. In light of these considerations, potassium intake should be modulated when its blood concentration reaches more than 5.5 mmol/l (86), especially in the stages G4 and G5 of CKD.

As food represents the main source of potassium (98), international guidelines recommend the use of nutritional strategies as an alternative to reduce potassium levels in CKD (84).

### Sources of Essential Amino Acids

The maintenance of nitrogen balance is essential for the preservation of a good nutritional state and optimal body composition. An important mechanism for maintaining nitrogen balance is represented by the supplementation of essential amino acids. A deficient dietary intake of amino acids increases endogenous nitrogen catabolism and the requirement of essential amino acids in the form of natural foods or pharmacological supplements. Based on these observations, an optimal dietary plan should be composed of a good balance of nutrients, especially amino acids derived from animal-source food with high biological value or vegetarian/vegan diets (e.g., normoprotein diet, vegan LPD, and sVLPD; 84).

## Management of Obese Patients With Chronic Kidney Disease

The close link between obesity and kidney disease demonstrates that weight loss is an important goal to be achieved by all obese patients affected by CKD and ESKD as weight loss can slow down the progression of CKD to end-stage disease, improve the outcome of renal transplant, and reduce the risk of death from CVDs. Unfortunately, there are no clinical practice guidelines to manage patients with obesity and CKD. For this reason, the dietary management of obesity in patients with CKD is based on a merger of clinical practice guidelines for the nutritional care of adults with CKD and the Italian Standard for the care of obesity. According to this evidence, optimal clinical dietary management in obese patients with CKD should be based on the Mediterranean or DASH diet scheme (87). In fact, the guideline for obesity care suggests the following strategy for weight loss:

- to realize a dietary plan, with controlled calorie restriction,
- to encourage regular physical activity,
- daily intake of whole grains and olive oil every day,
- five portions of vegetables and fruits per day,
- a portion of legumes two times per week,
- fish and fishery products as the main source of high biological value protein,
- to discourage the assumption of red meat, sugar, desserts, sugar beverages, processed meats, high sodium foods, and cheeses.

In the CKD stages G1–G3a, K/DOQI does not recommend specific amounts of energy intake, but recommends that energy intake levels support a balanced diet and maintain desirable body weight. For this reason, it is possible to realize low calorie and normoprotein diets. In the CKD stages G3b–G5, the recommendation is 35 kcal/kg/day for patients younger than 60 years and 30–35 kcal/kg/day for patients older than 60 years. This unavoidable condition makes calorie restriction impractical. Therefore, dietary treatment of obesity in the CKD stages G3b–G5 should be a qualitative revision of nutritional habits. Weight loss should be promoted through nutritional education and encouragement of regular physical activity (according to the patient’s possibilities; 99).

## New Perspectives in the Nutrition Field: Intermittent Fasting and Ketogenic Diets

### Benefits of Fast-Mimicking Diets in the General Population

In the management of obesity, caloric restriction (CR) is a necessary, although in many cases not sufficient, condition to induce effective weight loss. Indeed, other factors involved, such as macronutrient composition of the diet, metabolic adaptations, hormonal, and immune status, may affect both the extent and the quality of weight loss, with the selective loss of fat mass and the retention/increase of lean mass at the same time being the best desirable goal (100).

The MD represents to date the best evidence-based nutritional approach for the reduction of the cardiovascular risk and the incidence of metabolic disease (101), and is also ideal for the management of patients with CKD, given its nutraceutical and prebiotic properties (102). Anyway, it may not be the best option to induce weight loss. Indeed, its peculiar composition, with its caloric intake provided mainly by complex carbohydrates, and protein content derived mainly from vegetable and dairy sources and not exceeding 1 g/kg of body weight, could affect its efficacy in the extent and duration of weight loss. Moreover, the scarce amount of high-quality animal proteins needed to balance the muscle catabolism physiologically induced by CR, and the presence of high levels of carbohydrates, although complex, could not allow activating efficient lipolysis and fat mass reduction (103). Particularly, in settings of insulin resistance/altered glucose metabolism, conditions strictly interconnected with obesity, a high dietary carbohydrate/protein ratio is not favorable both from a metabolic point of view and in inducing weight loss (104, 99). Obese patients with CKD do not make an exemption. This is the reason why, when planning a CR, it is necessary to raise protein intake above the recommended value of 1 g/kg to spare muscle mass (103). The problem becomes more complex in the CKD setting, when protein restriction is usually required, especially in later stages. This concept finds support in a 2017 consensus of the Italian Society of Nephrology, stating the incompatibility between protein restriction and low-energy diets for overweight or obese patients with CKD (105).

To date, no guidelines focused on the management of obese/overweight CKD patients are available.

Evidence from studies on animal models and in the general population suggests that the use of nutritional approaches mimicking the physiology of fasting, such as ketogenic diets (KD) and intermittent fasting (IF), combined with CR, represent a promising strategy for weight loss, with additional cardio-metabolic benefits.

Ketogenic diets belong to the category of the low-carb diets (LCD). Indeed, they encompass a variety of nutritional schemes, which differ in their caloric and lipid content and are classified into low-calorie KD (LCKD), very-low-calorie KD (VLCKD; <800 kcal), and normocaloric KD. Weight loss is achieved by CR (LCKD or VLCKD), while normocaloric KD is used for other therapeutic purposes such as the management of drug-resistant epilepsy (106). Their common feature is minimal dietary carbohydrate intake (<50 g/day), with the aim of stimulating the physiological process of ketogenesis. It involves the mobilization of adipose-derived TG, their liver conversion into ketone bodies (KB; b-hydroxybutyrate and acetoacetate), and their distribution in the circulation: when ketonemia equals glycemia, the metabolic switch occurs, with KB becoming the preferred energetic substrate for the brain and other body tissues. The glucose-to-ketone switch is an evolutionarily conserved mechanism that allows survival during long periods of fasting by using KB as an alternative energy substrate. In the management of weight loss in obesity, when comparing classical CR with KD, the latter shows different advantages. It promotes a selective and massive depletion of fat mass, while sparing lean mass, and it allows better control of blood glucose and hyperinsulinemia and induces a greater satiety provided by KB that favors patient compliance with the dietary scheme (102, 101).

Beyond the effectiveness of KB as alternative fuels to glucose, they exhibit functional effects, by regulating the downstream expression of different strategic genes, such as sirtuins and genes signaling low energetic availability, thus shutting down anabolic pathways [such as mammalian target of rapamycin (mTOR) and protein kinase A as well as insulin and insulin-like growth factor 1 signaling] and upregulating catabolic/autophagy mechanisms (AMP-activated protein kinase, AMPK). Moreover, they activate antioxidant and cytoprotective pathways, resulting in DNA repair and reducing inflammation (107).

Intermittent fasting is a dietary strategy, whether or not used in combination with CR, which is characterized by specific food intake patterns, which differ in the timing when the food is consumed. The term “IF” usually encompassed different food intake schemes: alternate day fasting (ADF), 5:2 IF, or time-restricted eating (TRE). ADF consists of alternate days of fasting (complete or partial) and of *ad libitum* eating; in the 5:2 IF scheme, 5 days of *ad libitum* feeding are allowed, while on the remaining 2 days (consecutive or not), total calories are restricted (either completely or partially); the TRE scheme allows daily *ad libitum* food intake but temporally restricted in a timeframe of 4–12 h, depending on the protocols. The most popular is the 16:8 scheme with the fasting interval comprising evening/nocturnal hours (fasting from 4 pm until 8 am the next day; 108).

Intermittent fasting usually induces weight loss due to spontaneous reduction in calorie intake, as *ad libitum* hours/days of feeding often do not compensate the caloric reduction of the fasting intervals. Literature evidence do not demonstrate superior efficacy of IF in comparison with the classical daily CR in promoting weight loss in obese individuals (109, 110), although better outcomes on body composition parameters (fat/lean mass) were reported in those following IF (109).

Improved glucose metabolism with reduced fasting glucose and glycated hemoglobin, insulin sensitization through decreased fasting insulin and ameliorated homeostatic model assessment for insulin resistance index is the first to be reported (107, 110, 111). Moreover, improved BP control (107), loss of abdominal fat (109, 107), and a reduction of inflammation have been ascribed to IF (107, 108). Very interesting data come from studies on animal models, where the application of different IF patterns resulted in the regulation of a complex network of genes, dysregulated in animal models of obesity (108). These genes include those related to the biological circadian clock, inflammatory/oxidative pathways, mitochondrial autophagy mechanism and repair mechanisms, modulation of the autonomous nervous system and the immune system (107, 108), making this pattern attractive as a therapeutic option in humans, although evidence from large trials is limited. In animal models, the fasting-mimicking diet, a dietary protocol involving strict and short-term CR combining with a good fat nutritional composition, demonstrated its benefits in reducing the local and systemic inflammation in the context of intestinal bowel disease, while in diabetes type 1 and 2, it was shown to induce β-cell regeneration and insulin secretion, promoting glucose balance (108). Moreover, some evidence suggests that the benefits of fasting at the metabolic level can be mediated by gut microbiota (112), which in turn is modulated by IF that increases its diversity and enhances antioxidative microbial metabolic pathways (107). The microbiota itself shows circadian fluctuations that are deranged in animal models of obesity and can be restored by TRE (113).

The functional mechanism of IF relies on the alternation of the glucose-to-ketone switch and *vice versa*. In the first phase, coinciding with the fasting interval and in a way similar to KD, all the feeding-mode pathways are switched off: mTOR is downregulated with subsequent protein synthesis inhibition, while sirtuin is induced and autophagy is stimulated. On the contrary, in correspondence to the refeeding phase, the nutrient-depending pathways are switched on: mTOR and protein synthesis are reactivated, thus allowing repair and organules/cell/tissue regeneration (107).

### Application of Ketogenic Diets/Intermittent Fasting to Chronic Kidney Disease: Available Data and Future Perspectives

In the context of CKD, only a few studies on fast-mimicking diets are available.

Some authors hypothesize beneficial effects on the kidney function by LCD/KD due to the prominent weight loss associated with the reversal of hyperinsulinemia, which is known to be linked to kidney damage by several mechanisms. Apart from its association with overweight/obesity, hyperinsulinemia is known to induce elevated BP by increasing renal angiotensin receptors and endothelin (114). In this regard, the group of Mitchell performed an interesting study on 2,000 patients undergoing LCDs, retrospectively categorized into CKD stages 1–3. This study shows the safety and the advantage of KD in patients with CKD at intermediate stages 2–3 with ameliorated eGFR, after weight loss with this approach. Anyway, the same result was not observed in patients with CKD stage 1, showing a worsening of eGFR after nutritional treatment. This result probably suggests that in intermediate stages of CKD the advantages of a rapid and important weight loss outbalance the risk of renal function decline observed in early stages, probably through the amelioration of BP and glycemic control. The main limitations of this study are the absence of an intervention and its retrospective nature (114).

Consistently, the advantages of a significant weight loss on renal function are well assessed (99, 114). This has been demonstrated in the context of bariatric surgery, where a reduction in the risk of proteinuria/albuminuria and an amelioration in eGFR are observed, probably due to the massive weight loss induced rather than the surgery *per se* (114).

The most pronounced beneficial effects of LCD are observed in the context of diabetes, where they promote reductions in glycated albumin and glycemia. Moreover, increased protein intake associated with LCD shows a 14% less probability of incident or progressive CKD and diabetes, while, in contrast, a major carbohydrate intake is associated with a 15% increased probability of incident or progression of CKD (99). It is worth noting that in subjects with an eGFR > 80 mg/ml/1.73 m^2^ a high protein intake is not associated with a decline in eGFR (115, 99), differently from subjects of KDOQI CKD stage II or more (99).

No medium- and long-term data are available on the risk/benefit ratio of LCD in CKD. Anyway, our studies agree with some authors on the potential usefulness of KD, to be used in intermediate stages of CKD to slow disease progression, as a tool for rapid weight loss, effective in the management of obesity, especially if associated with diabetes or hyperinsulinemia (99, 114). Contraindications to VLCKD generally include kidney failure and CKD. For this reason, accurate clinical evaluation of eligible patients, with a particular attention to the hydration status and electrolyte balance, should be performed by the nephrologist, so that if the advantages of rapid and massive weight loss outbalance the clinical risk, a short supervised cycle of VLCKD can be allowed. Actually, the suitability of VLCKD in the context of CKD at different stages is reported in the review of Lambert, offering practical guidance on the application of this dietary strategy using food substitutes, underlining feasibility and safety, along with the need for personalization according to renal disease and strict medical control (116).

We also report two studies by the same group of researchers, in which an unconventional, LC, and high-protein weight loss approach was applied in a small cohort of patients with dialysis and in a case study of a patient with advanced CKD. In the first study, the LC approach was combined with incremental dialysis sessions, showing, beyond a considerable weight loss, also an amelioration of calcium–phosphorus–PTH metabolism and a reduction/discontinuation of phosphate binders, erythropoietin, and antihypertensive drugs (117). In the second case study, a patient with advanced CKD underwent the same approach that allowed him on the transplant wait list: he lost weight and fat mass, improved lean mass and hydration status, and stayed free from dialysis for over a year after the discontinuation of the diet. The authors hypothesize that the rapid increase in proteinuria and a decrease in kidney function that occurred immediately after starting the first phase of the diet were due, respectively, to the discontinuation of the previous 0.6 vegetable ketoacid-supplemented diet and to the strong sodium restriction foreseen by this specific dietary protocol. Indeed, the kidney function stabilized after the discontinuation of the diet. These two reports evidence, as the authors also state, the feasibility and safety of dietary approaches usually proposed to obese people with non-CKD, even in the context of dialysis, where weight loss is regarded as inappropriate (as distinctions between intentional and unintentional weight loss are often not made). We agree with the concept that approaches different from the classical LPD in CKD should not be banned *a priori*, provided that an accurate and personalized evaluation of the risk/benefit ratio and a strict nutritional, clinical, and laboratory control are carried on. As already reported, weight loss and protein restriction are conflicting (105). Thus, depending on the clinical evaluation, and based on the risk/benefit assessment, we propose that priority to one of the two should be given by the nephrologist in a given phase, provided that nutritional therapy can be switched between phases of rapid weight loss and phases of weight and kidney function stabilization. Moreover, the results comparable to those of the two reported studies could probably be obtained with the use of less extreme, personalized, and sustainable approaches (no need for total sodium exclusion or sustainable food combinations, without dissociation), targeted to preserve lean mass along with allowing fat mass to decrease. Furtherly, the LC approach could be used for a shorter period of time (4–6 weeks) and cycled with a low-protein/Mediterranean approach to increase adherence and mitigate the renal load of increased protein intake.

Finally, beyond weight-loss effects, there is evidence in the literature of a potential therapeutic use of KD in CKD for its functional effects. In an animal study, a 2-month KD has been shown to completely reverse diabetic nephropathy, with an improvement in glomerular histopathology (118). In the context of polycystic kidney disease, defective glucose metabolism and glycolysis-dependent cystogenesis have been demonstrated (119). Preclinical models show that both KD and IF are effective in inhibiting the mTOR/AMPK pathway, cystogenesis, and renal fibrosis, and that these effects are specifically induced by ketosis, as demonstrated by the inhibition of PKD progression by the administration of b-hydroxybutyrate (120).

The literature regarding “IF and CKD” is restricted to a few studies, which exclusively report the outcomes in the Muslim population undergoing Ramadan. No specific trials applying IF/TRF in CKD are available.

In their brief communication, the group of Mbarki reports that in a prospective study of 60 patients with CKD who were allowed to fast during Ramadan, after excluding all medical contraindications [severe or resistant arterial hypertension, insulin-requiring diabetes, acute renal failure (ARF), active renal disease, recurrent urolithiasis, or terminal chronic renal failure], 11.7% of them developed superimposed ARF and that this risk was significantly associated with a higher CKD stage (eGFR < 60 ml/min/1.73 m^2^). In these patients, hypotension due to dehydration was also more frequently observed (121). In a study by El-Wakil, the central role of hypovolemia in determining renal tubular lesions in this setting is evidenced: the authors explain that renal tubules are particularly vulnerable to renal hypoperfusion, and the normalization of renal function after the end of fasting supports the hypothesis that renal failure in fasting is mediated by imbalances in water and electrolyte intake (122).

In a prospective cross-sectional study by Adnan of 535 Muslim patients on hemodialysis, Ramadan fasting is reported to have beneficial effects, being associated with weight reduction, improved serum albumin, and phosphate (123).

A 2019 literature review reports different and conflicting results emerging from different studies on the effects of fasting during Ramadan in CKD, HD, and transplanted patients. Indeed, in patients with CKD, some studies report negative outcomes during fasting. Beyond the already reported ARF and hypotension (121, 122), some authors report a reduction in eGFR and an increased incidence of cardiovascular events (especially in people with preexisting CVD), particularly in those patients with severe CKD (eGFR < 35 ml/min/1.73 m^2^).

The main limitations of studies in the field of IF and CKD are the observational nature of the studies and the great heterogeneity of results, the study populations, the small sample size, and subsequently observed outcomes. Currently, there is a lack of guidelines for patients with CKD willing to fast during Ramadan. Overall, Ramadan fasting appears to be safe in both transplanted and in patients with intermediate stage CKD. Worsening renal function and the incidence of negative outcomes seem to be associated to the severity of the stage of renal disease (124). In CKD stages 4 and 5 and in patients with dialysis, medical supervision and monitoring of water and electrolytes (especially potassium and phosphorus) are mandatory (125, 124).

Ramadan fasting can be considered on the IF spectrum, with a substantial difference: it does not allow food, but even water from sunrise to sunset. In contrast, schemes such as 16:8 TRF allow free water intake and even small quantities of insulin-response neutral foods (i.e., fresh vegetables). This difference can be substantial in patients with CKD, where many of the reported adverse effect seem to be mediated by hypovolemia and electrolyte imbalances due to water restriction. As highlighted above, no trials with IF/TRF in the CKD population are available.

Anyway, based on the beneficial effects reported in the literature in the general population and in CKD under medical supervision, we will attempt to speculate on the potential advantages of applying fast-mimicking diets in CKD. Firstly, the improvement of glucose metabolism (with a reduction of circulating glucose, insulin, and leptin levels) is favorable in obese subjects with CKD, often characterized by insulin resistance.

Given the strong need for adequately powered RCTs evaluating beneficial outcomes and adverse events in obese patients with CKD at different stages, we speculate here on the advantages of using KD and IF in clinical practice, particularly in intermediate stages of CKD. Especially for KD, due to its potential adverse effects (arrhythmias, transient elevation of blood uric acid, electrolyte imbalances due to massive urinary loss of cations, and risk of gallbladder stones), the choice of patients eligible for KD should be done accurately, also depending on their acceptance of the dietary scheme.

In our opinion, the advantages of fast-mimicking diets could be applied to obese patients with CKD in whom classical CR approaches are not successful, provided that some mandatory conditions are respected:

1. an evaluation of clinical conditions has been carried on to exclude potential contraindications,
2. adequate patient selection has previously been made to guarantee high adherence to the dietetic treatment,
3. the dietary treatment is accurately tailored to the patient, and
4. the dietary treatment is carried out under strict medical control.

These tools could be used in short-duration cycles of 4–8 weeks, which would be interchanged with classical, normocaloric (and low-protein, if necessary) maintenance approaches in Mediterranean style. Weight loss, in general and particularly in the patient with CKD, should be a personalized step-by-step process, strictly supervised and designed in a way that could be sustainable for the patient.

## Conclusion

Nutrition represents a modifiable risk factor for frailty, and as such, is a target for both prevention and treatment of this debilitating condition. However, to design effective dietary interventions, a clearer understanding of the key dietary components and underlying mechanisms of action is needed. Progress in understanding the role and importance of nutrition and to address how diet can be used for the prevention and treatment of frailty requires a multidisciplinary approach. Therefore, a collaborative group of experts from the fields of nutrition and medicine, from a variety of backgrounds, will work together to promote a dialog to address the potential of diet within strategies to prevent and treat chronic illnesses and frailty.

## Author Contributions

AS contributed to conceptualization, writing, and editing of the manuscript. CC and GC contributed to the conceptualization and drafting of the work. GC conceived the figure. MTC, RP, PMA, and GR contributed to the draft. LG contributed substantially to the reported work through critical revisions and draft editing. All authors read and agreed to the published version of this manuscript.

## Conflict of Interest

The authors declare that the research was conducted in the absence of any commercial or financial relationships that could be construed as a potential conflict of interest.

## Publisher’s Note

All claims expressed in this article are solely those of the authors and do not necessarily represent those of their affiliated organizations, or those of the publisher, the editors and the reviewers. Any product that may be evaluated in this article, or claim that may be made by its manufacturer, is not guaranteed or endorsed by the publisher.
